# Supplementary material for: Derivation and simulation of a computational model of active cell populations: How overlap avoidance, deformability, cell-cell junctions and cytoskeletal forces affect alignment
Source: PLoS Comput Biol. 2024 Jul 29;20(7):e1011879. doi: 10.1371/journal.pcbi.1011879 (PMC11309491; doi:10.1371/journal.pcbi.1011879)
Supplement: Attachment — Submitted filename: answers_to_reviewer_comments.pdf [file pcbi.1011879.s005.pdf]

Reviewer Comments

Vivienne Leech[1], Fiona N Kenny[2], Stefania Marcotti[2], Tanya J Shaw[2], Brian M Stramer[2], and Angelika Manhart[1,3]

[1]Department of Mathematics, University College London, London, UK

[2]Randall Centre for Cell and Molecular Biophysics, King's College London, London, UK

[3]Faculty of Mathematics, University of Vienna, Vienna, Austria

May 7, 2024

1 Reviewer 1

Reviewer: *The authors propose an ellipsoidal cell agent-based model (ABM) for cells interacting in 2D with avoidance of overlap. The model is formulated as an energy a minimization. The aspect ratio of cells, their elastic (head-tail) tethering, and forces due to supra cellular actin cables are considered. They find that self-propulsion, along with these mechanisms aid affects the cells' ability to align with neighbours. The higher aspect ratio leads to higher alignment and packing of the cells. The paper is well-written and interesting. I liked the step-wise approach, where the simplest cases are treated first, and then new features are introduced. I enjoyed reading it. That said, there are a few ways to improve its readability.*

Reviewer Comment 1: *The authors approximate cells as ellipses. How much does this lose? Actual cells can slightly rearrange their shape to avoid having to turn or align, no? Would the conclusions hold for cells that deform by pseudopod retraction/protrusion in random directions, not maintaining exact ellipsoid shape?*

Response 1: We agree that the model assumption of approximating cells as ellipses doesn't capture the full complexity of observed cell shapes. Our goal was to focus on two main aspects: that cells are elongated and that they can deform. We argue that over the timescale we are interested in, most elongated shapes (other authors have e.g. used capsule shapes or similar) would capture the observed cell shapes without going into the extra level of detail and complexity. Our model strategy also allows for relatively straightforward extension to other cell shapes, however this is beyond the scope of this work. We have added some comments about this and how to include pseudopod retraction/protrusion in random directions in the text.

Reviewer Comment 2(a): *The authors suggest that (L 76-77) CPM are costly compared with other types of models. In my experience this is not the case, and there are free platforms like Morpheus that make CPM simulations very easy (and also very reproducible). As far as I could see, only the actin cables would be tricky to include. I wonder if the model conclusions would be robust to other platforms like that.*

Response 2(a): Thank you for pointing this out, we have edited our statement about these types of models in the text. We assume that several conclusions would hold also for CPM simulations and that e.g. the plots in Fig. 2 would be similar. We'd be curious whether CPM would also give an optimal ratio of self-propulsion to overlap avoidance (Fig. 3) and whether CPM would predict that shape deformations help alignment (Fig. 5). A collaboration with a CPM-experienced group in the future would be interesting.

Reviewer Comment 2(b): *This brings up the question of whether the model is purely deterministic? Would a little noise help or hinder alignment? [Note that CPM has an inherent energy that can be defined to avoid or promote cell-cell contact. It also has some inbuilt stochasticity. Directed cell motility can also be implemented as can a kind of preferred length and perimeter to area ratio.]*

Response 2(b): We agree that the effect of noise on the model is something missing from the paper. We have therefore incorporated orientational noise into the model and added a paragraph on the effect of this in the appendix. We have also pointed out other ways in which noise could be included in the model in the main text.

Reviewer Comment 3: *The paper should cite the work of Eirikur Palsson, who has a population of deformable ellipsoidal cells model with forces due to cell overlap in 3D. (He was not studying alignment, but the general idea is related.) There are also some CPM simulations with cell alignment in the literature and a free example in Morpheus that should be cited in case not already done.*

Response 3: We thank the reviewer for this suggestion and have cited these two works in the text.

Reviewer Comment 4: *Model presentation and equations: In general, the equations could be explained a little more clearly. Note that equations were not all numbered, so it's hard to refer to them. For example, Section 2.2 is a bit "formal" and could use a couple of sentences for biological readers. A small panel in figure 1 with your parametrization might help the non mathematician understand your energy terms better.It would also be nice to have one (or more) small panels in some figures showing the case of 2 cells where the quantities in ODEs are labeled. While I understand that the paper is mainly directed to modellers, the current formalism presents a barrier to readers with less formal background, so a few more diagrams or text explanations could help.*

Response 4: We thank the reviewer for their suggestions and have implemented all of the above. We hope that the addition of the extra diagrams and explanations will make it easier for readers with a less formal background to follow.

Reviewer Comment 5(a): *Can the authors comment on how different is this mechanism from simple repulsion between centroids of cells? Their work probably allows some insights about the connection.*

Response 5(a): We thank the reviewer for their suggestion and have added some further interpretation of the model, highlighting how it varies from a model of simple repulsion between centroids. The main difference is that a model with simple repulsion between centroids would only account for changes in position, not changes in orientation. If there is no change in orientation as a result of cell interactions, the model would be missing any sort of alignment mechanism.

Reviewer Comment 5(b): *Would cell-cell junctions along the sides help alignment? I.e. can the geometry of junction placement help with alignment?*

Response 5(b): We thank the reviewer for this suggestion and have included an additional study in the appendix exploring the effect of the placement of cell-cell junctions on alignment. We have also noted an alternative modelling approach that could be taken to model cell-cell junctions being able to form anywhere round the cell.

Reviewer Comment 5(c): *Is there a way to compare the model predictions with experimental observations? To quantify alignment and aspect ratios etc? Please correct me if I am wrong, but so far it appears that the experiments are just to show some images of cells. There is not yet any preliminary representation of the cells by ellipsoids or the alignment of those cells. How hard would that be? While this seems to be called "future work" is some preliminary comparison feasible?*

Response 5(c): Some preliminary qualitative conclusions from comparing the model to experimental data can be found in the appendix of [Kenny et al., 2023]. We have not included further details in this paper since this is intended to be a computational study of the model motivated by experimental evidence, however we have cited the findings in [Kenny et al., 2023] in the text.

Reviewer Comment 5(d): *The authors suggest a number of interesting future directions. One of these, namely doing the 2-cell stability analysis would seem to belong in this paper, to show some small analytical treatment. Other aspects are fine for later work.*

Response 5(d): We agree that this could fit naturally into the end of this paper, however upon working through the analytical results, even the 2-cell stability analysis involves the technical treatment of degen-

erate limits. We will therefore address this, along with a number of other analytical results, involving asymptotic methods, stability analysis and consideration of long term behaviour in a separate paper that will be submitted within the coming months.

Minor issues

- *Fig 1 - what are the green and magenta tracers marking?* They are marking different cells, one is labelled in green while all the others are labelled in violet. This allows for cell overlap to be investigated.
- *Is X_i the cell centroid?* Yes
- *The small case x is not fully defined. Should it also have a subscript for cell i?* Thank you for this comment, we have defined it in the text.
- *"leading to the area elements abs." - unclear* We have added some clarification on how this arises in the text.
- *a self-propulsion speed that is independent of friction. [Is there evidence for this]?* If we were comparing directly to experiments, this would have to be specified (and should be motivated by experimental data). Here, we work in a non-dimensional setting, where all parameters are (non-dimensional) ratios of physical parameters. Therefore the results are the same, whether self-propulsion is independent of friction or not; it merely affect the interpretation of the non-dimensional parameters. We have added a comment addressing this.
- *Will cells always move in a direction of their orientation or be pushed in the direction of greatest change in overlap?* This happens both at once and will depend on the value of ν.
- $E_{overlap}$ *- not clear what V is, and what abs refers to. Please mention that V will be discussed later.* Thank you for pointing this out, we have made this clearer in the text.
- *L 149 We therefore choose V to be constant with value σ in regions of overlap and zero elsewhere. [How hard would it be to have V depends on the contact length or overlap area?]* V is a potential whose effect is punishing any cell overlap. The larger the overlap area, the greater this effect is. Cells move to reduce this overlap. Therefore V does already incorporate overlap area. One could have other forms of V where e.g. V depends explicitly overlap area or contact length (introducing a memory effect), however computations would likely be much more involved. Including any such non-local effects into the potential V would massively increase the complexity.
- *L 160 "We see that the cell's center is being pushed in the direction normal to the vector connecting the points of overlap." [Surely the model was designed this way, so it would be nice to say this earlier as a motivation for the model equations, and then confirm it here.]* The beauty of the modelling is

that we did not design it this way! The modelling equations are a result of only defining the potential and asking how cells need to move to minimise the energy.

- *L 214 For one cell we obtain the following equation for how the aspect ratio r changes over time: [explain where from, and why aspect ratio should change if this is one cell with no interactions.]* Thank you for pointing this out, we have made this clearer in the text.
- *Fig 4 " Single NDF (A) and KDF".. I suggest not using abbreviations in this figure for better readability.* Thank you, this has been changed.
- *L 228 suggesting that shape changes aid alignment. [Strictly speaking, this applies to changes in the aspect ratio, so unless you can assert that it is the same as arbitrary shape changes, perhaps soften the wording?]* We agree and have softened the wording here.
- *L 243 This suggests that allowing cells to deform dynamically as a reaction to overlap introduces a more flexible way to use the available space leading to higher alignment. [Can you support this statement with some text about what "more flexible" means?]* Thank you, we have added some additional explanation to make this clearer in the text.
- *When cells change shape do you preserve the area (exactly, or approximately)?* The area is preserved exactly.
- *L 293 junctions hinder alignment, because they lead to cells forming clumps where more than two cells are joined at one point [is this a model artifact?]* Yes, it is a result of the model and the cell geometry. We hope that adding a study on the effect of cell-cell junctions forming at the sides demonstrates this further.
- *Fig 8 - I like the idea of representing the cells as a graph, but why only linear chains? Why not some branching graph?* A good question! We suspect, if the junction range was higher or the junctions are stronger, we would see more branches. Otherwise the self-propulsion probably breaks up non-linear chains.
- *L 329 the derived equations strike a useful balance between being complex enough to capture the desired phenomena, while being simple enough to be interpretable. [Can you comment on how much analysis can be done? Why not treat one of the simplest cases here?]* We have added some further comments on what the analysis involves and why we do not include it in this paper.

2 Reviewer 2

In this paper, the authors model the collective alignment of cells by incorporating mechanisms of self-propulsion and overlap avoidance. They also investigate the effects of cell deformability and cell-cell junction

formation, comparing the results to experimental measurements. Overall, the paper is well-written, and the findings appear intriguing.

Reviewer Comment 1: *The authors present the model as a process of energy minimization per time step, but it remains unclear why this approach is chosen over defining the physics of the system through a force balance equation. Utilizing a force balance equation would provide a clearer physical principle, where frictional forces balance forces derived from a potential and active forces. This approach would likely yield the same dynamical equations, with the energy dissipated as heat.*

Response 1: We have added this alternative derivation into the appendix for readers to refer to if this method is preferred.

Reviewer Comment 2: *In the simulations, we observe lateral sliding of cells (perpendicular to the direction of propulsion) due to pushing forces from neighboring cells. However, it's questionable whether this behavior accurately represents cell movements during alignment. Cell movement typically involves crawling along surfaces through protrusions, adhesion formation, and contractions. While the model captures some aspects of cell propulsion, it's unclear how the perpendicular sliding observed in the simulations translates to real-world scenarios.*

Response 2: We argue that the mechanism that could cause the cells to move in a perpendicular direction captures a whole array of behaviours that cells could exhibit to avoid overlap. In the experimental video which can be found in [Kenny et al., 2023] we can see cells exhibiting this behaviour to avoid overlap. We have referenced this video in the text to point readers towards an example of cell behaviour that can be related back to the model.

Reviewer Comment 3: *The paper includes a comprehensive list of references to related works in active and motile cell systems, primarily in the introduction. However, I find a notable absence of meaningful connections between the results of this study and prior research in the broader field of polar active matter. Previous works have investigated the stability properties of homogeneous polar and nematic states, as well as mechanisms of defect formation. It's essential to contextualize the specific findings of this paper, particularly the basic model presented in Fig. 2, within the existing literature to justify the novelty and accuracy of the results.*

Response 3: We have added further citations and connections between our results for the base model and existing results from other models in the field of active matter to give some additional context.

Minor Comment: *The color scale in Figures such as 2A should ideally cover the range from 0 to 2pi.* The colour scale ranges from 0 to pi because we hoped to show the nematic alignment in the snapshots. We have changed the labelling of the colorbar from 'orientation' to 'nematic orientation' to make this clearer.

3 Reviewer 3

The article by Leech et al. describes a well designed study on agent-based simulation of fibroblast alignment observed during wound healing. The article utilizes a propelled particle approach, and considers mechanical contacts between cells. In the 2nd half, the model incorporates the possible shape changed by the cell and potential role of actin cables. Overall, I think the paper is well organized and clearly written. However, there are no compelling results. The model formulation lacks some fundamental elements such as random forces. I would like the authors to address the following main conceptual questions.

Reviewer Comment 1: *The article uses a propelled particle approach. This is the most popular approach in literature, first was used by Vicsek et al. I understand that most people take it for granted that this applies to cells. In actuality, this is not too clear. In fact, it was shown pretty convincingly that cells move according to a persistent random walk model, often biased by ECM fibers (Wu et al. PNAS 2014). The next question is whether the persistent random walk model is equivalent to the propelled particle model. I think it can be shown pretty convincingly that this is not the case. I think the authors should at least mention that other models of cell movement have been proposed that are potentially fundamentally different.*

Response 1: We thank the reviewer for these suggestions and have pointed out persistent random walks as an alternative modelling approach for cell motion.

Reviewer Comment 2: *In the context of persistent random walk model, its application to multicellular case in confluent epithelium case has been explored by Li & Sun, Biophys J. 2014 and Koride et al, APL Bioengineering 2018. These papers use the vertex model, which describes cell shape changes crudely, but nevertheless allows for some sort of mechanical interaction. I'm not sure how this compares with the elliptical approach, but should be discussed.*

Response 2: We thank the reviewer for these suggestions and have added references to these and discussed vertex models in more detail in the introduction.

Reviewer Comment 3: *All of the models mentioned above utilizes some sort of random force. In other words, cells motion direction will randomly diffuse, ultimately generating diffusive behavior. This observation has been well established experimentally. There is no random force discussed in this work, and therefore not surprising that we see alignment. Diffusive behavior of cell motion is pretty well established, the lack of which*

in this work is a major question mark. I understand that authors have cell-cell interaction through actin dynamics and cell shape change, but these are not random forces. I worry that the author is missing a major ingredient.

Response 3: We thank the reviewer for these comments. We have added a section into the appendix which explores the effect of orientational noise on alignment. We would argue that it is not necessarily obvious that we get alignment out of the model, even without noise, since alignment isn't an explicit model ingredient. We agree that further noise could be added into other aspects of the model, however this would add extra parameters with the risk of not learning much else about the model. We have mentioned that adding noise into each model component could be an option which could make the model align more closely with the biology in certain contexts, something we have explored in [Kenny et al., 2023] and referenced in the text.

Reviewer Comment 4: *The cell shape change model is reasonable but the actin cable model is not. Actin stress fiber formation is dynamic and stochastic, as has been shown extensively in biological literature. Modeling of actin stress fiber is also available. Here the authors model actin fibers as springs and rods, with even bending contribution. This is not aligned with biological observation, which shows that actin stress fibers turn over on the time scales of minutes, and dissipate and reform. Actin cytoskeleton on the time scale of cell movement is liquid like. This type of dynamics could well generate random forces that ultimately describes cell dynamics observed in this context.*

Response 4: We have added citations to more detailed models of actin stress fibres. In this work we do not aim to describe actin bundles within each cell, but rather focus on the effect of transcellular actin bundles on the orientation of neighbouring cells. The dynamics and forces at the cell-cell junctions are created by the interplay between actin, accessory molecules and cell-cell junction components and are likely dynamic and complicated. We chose to model the cell-cell junctions (and not actin itself) as elastic springs. The biological observation of rather straight transcellular actin bundles suggest communication between the cytoskeleton of connected cells. This could be mediated by an actin bending stiffness (which is how we derive our equation) or e.g. cells pulling on each others cytoskeleton. In both cases the effect would be alignment of cell orientation, which is what our model describes. In the future it would be interesting to start with detailed stress fibre models and compare the effects.

Reviewer Comment 5: *Along the same vein, cell-cell junctions can also dynamically form and break, generating friction-like behavior between cells. Cell-cell junction should not be modeled as static linkages.*

Response 5: We agree that in reality cell-cell junction formation and breaking is a lot more dynamic. We have added some sentences in the text when introducing the cell-cell junction model to address this.

The goal of our study is an initial exploration into the effect that cell-cell junctions can have on collective alignment. We appreciate that our deterministic representation of cell-cell junctions present a simplification. A detailed stochastic model would lead to the inclusion of several more parameters. To measure these we would require detailed imaging data of cell-cell junctions over time. Further, parameter exploration into general model behaviour would become very challenging and computationally costly. We therefore believe our model assumptions strike the right balance for studying the overall effect of cell-cell junctions in the context of collective dynamics.

References

[Kenny et al., 2023] Kenny, F. N., Marcotti, S., De Freitas, D. B., Drudi, E. M., Leech, V., Bell, R. E., Easton, J., Fleck, R., Allison, L., Philippeos, C., et al. (2023). Autocrine il-6 drives cell and extracellular matrix anisotropy in scar fibroblasts. *Matrix Biol*, 123:1–16.
