# Supplementary material for: Derivation and simulation of a computational model of active cell populations: How overlap avoidance, deformability, cell-cell junctions and cytoskeletal forces affect alignment
Source: PLoS Comput Biol. 2024 Jul 29;20(7):e1011879. doi: 10.1371/journal.pcbi.1011879 (PMC11309491; doi:10.1371/journal.pcbi.1011879)
Supplement: Attachment — Submitted filename: answers_to_reviewer_comments2.pdf [file pcbi.1011879.s006.pdf]

Reviewer Comments

Vivienne Leech[1], Fiona N Kenny[2], Stefania Marcotti[2], Tanya J Shaw[2], Brian M Stramer[2], and Angelika Manhart[1,3]

[1]Department of Mathematics, University College London, London, UK

[2]Randall Centre for Cell and Molecular Biophysics, King's College London, London, UK

[3]Faculty of Mathematics, University of Vienna, Vienna, Austria

July 9, 2024

Comment from Reviewer 2. The authors have addressed my comments, which improved the manuscript. However, the connection between the results of this study and prior research in the broader field of polar active matter remains weak (comment 3 of my first review). Given extensive work on related systems (e.g. see review `https://doi.org/10.1103/RevModPhys.85.1143`), I recommend that the authors further improve this connection.

Answer to Reviewer 2. Thank you for that comment. To strengthen the connection with the field of polar active matter we have: 1. Added a reference to the review paper in the introduction and discussed how energy input on the local scale pushes the system out of equilibrium. 2. We have added a dedicated paragraph with at the end of Section 2 which discusses our model and its results in relation to the current state of the art of the field of polar active matter. In particular we discuss how our model should be classified and how self-propulsion in previous and our work changes nematic order. For aspects we did not investigate in this work (phase transitions and orientational defects) we discussed the literature. Finally we mentioned the connection to continuum models of active matter and how they can enhance our understanding of polar active matter, such as understanding the stability of steady states.
